# *In Vitro* Antioxidant Activity Potential of Lantadene A, a Pentacyclic Triterpenoid of *Lantana* Plants

**DOI:** 10.3390/molecules170911185

**Published:** 2012-09-19

**Authors:** Chong Grace-Lynn, Ibrahim Darah, Yeng Chen, Lachimanan Yoga Latha, Subramanion L. Jothy, Sreenivasan Sasidharan

**Affiliations:** 1 Institute for Research in Molecular Medicine (INFORMM), Universiti Sains Malaysia, Pulau Pinang 11800, Malaysia; Email: gracelynn87@gmail.com (C.G.-L.); latha_usm@yahoo.com (L.Y.L.); jothylachumy@yahoo.com (S.L.J.); 2 School of Biological Sciences, Universiti Sains Malaysia, Minden 11800, Penang, Malaysia; Email: darah@usm.my; 3 Dental Research & Training Unit and Oral Cancer Research and Coordinating Centre (OCRCC), Faculty of Dentistry, University of Malaya, Kuala Lumpur 50603, Malaysia; Email: chenyeng@um.edu.my

**Keywords:** antioxidant activity, free radical, lantadene A, triterpenes

## Abstract

Lantadenes are pentacyclic triterpenoids present in the leaves of the plant *Lantana camara.* In the present study, *in vitro* antioxidant activity and free radical scavenging capacity of lantadene A was evaluated using established *in vitro* models such as ferric reducing antioxidant power (FRAP), 2,2-diphenyl-1-picryl-hydrazyl (DPPH•), hydroxyl radical (OH•), nitric oxide radical (NO•), superoxide anion scavenging activities and ferrous ion chelating assay. Interestingly, lantadene A showed considerable *in vitro* antioxidant, free radical scavenging capacity activities in a dose dependant manner when compared with the standard antioxidant in nitric oxide scavenging, superoxide anion radical scavenging and ferrous ion chelating assay. These findings show that the lantadene A possesses antioxidant activity with different mechanism of actions towards the different free radicals tested. Since lantadene A is a very popular drug in modern medicine, it is a promising candidate for use as an antioxidant and hepatoprotective agent.

## 1. Introduction

**Figure 1:**
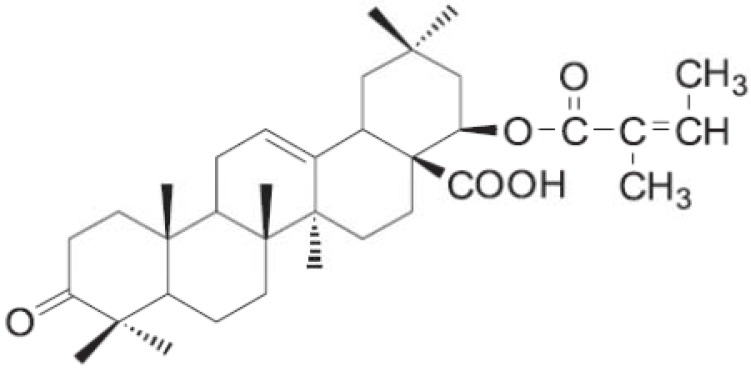
Lantadene A.

Triterpenes are a group of widespread natural compounds containing six isoprene units with the basic molecular formula, C_30_H_48_. They are synthesized in many plants by the cyclization of squalene [1]. They are obtained from plants and especially from bark of trees such as plane, cork and birch but are also found in liquorice roots where they are particularly abundant. The most studied triterpenes are the pentacyclic triterpenes as their biological properties are considerable [2]. Pentacyclic triterpenes have contributed to the development of several modern therapeutic drugs. For example, triterpene acids (betulinic acid and ursolic acid) have been shown to exhibit significant anticarcinogenic and anti-HIV activity [3,4], while lupeol is a competitive inhibitor of both trypsin and chymotrypsin [5] and the antiphlogistic activity of betulin was confirmed in various experimental models [6]. α-Amyrin and β- amyrin have been patented for use in the cosmetic industry as hair and skin protecting agents. Betulin and its derivatives (esters and ethers) have been proven to have an antinociceptive effect against visceral pain in mice produced by intraperitoneal injection of acetic acid [7]. Lantadenes are pentacyclic triterpenoids present in the leaves of the plant *Lantana camara* [8,9,10]. Previous studies showed that ingestion of lantana (*Lantana camara*) foliage by grazing animals causes hepatotoxicity and photosensitization [9,11]. However, there is no detail study on the possible hepatoprotective activity of lantadene A (Figure 1) which is widely used in the development of modern therapeutic drugs. Moreover, pentacyclic triterpenes is now being marketed as therapeutic agents, and a couple of synthetic pentacyclic triterpenes derivatives are now undergoing clinical trials. 

Pentacyclic triterpenes constitute an important group of natural compounds. However, previous study also showed that ingestion on lantadene induced hepatotoxicity in guinea pigs and sheep. There has been no consensus on the identity of the lantana toxins. Some research groups have reported lantadene A to be the hepatotoxic principle, while others provided evidence that pure lantadene A did not elicit hepatotoxicity. The action of lantadenes associated with hepatoprotective actions or hepatotoxicity is contradictory due to the presence of antioxidants found in the plant. There is no detailed study reporting on antioxidant activity of lantadene A. Therefore, there is a need to evaluate and verify this activity, and the current study was thus initiated to investigate the antioxidant activity with various *in vitro* assays.

## 2. Results and Discussion

### 2.1. DPPH Radical Scavenging Activity

**Figure 2:**
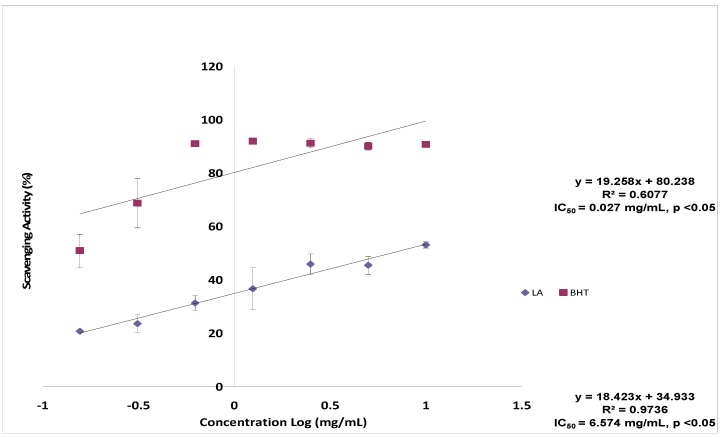
Scavenging effect of lantadene A on DPPH free radicals compared with butylated hydroxytoluene (BHT).

In this study, lantadene A showed moderate DPPH radical scavenging activity. Figure 2 depicts a steady increase in the percentage inhibition of the DPPH radical by the lantadene A up to a concentration of 0.4 mg/mL, after that there was a leveling off with much slower increase in inhibition. The maximum DPPH radical scavenging activity of lantadene A was 53.11 ± 1.14% and the IC_50_ was 6.574 mg/mL (Figure 2). Compared with the reference BHT (IC_50_ = 0.0270 mg/mL), lantadene A showed lower activity on DPPH. The DPPH assay is often used to evaluate the ability of antioxidants to scavenge free radicals which are known to be a major factor in biological damages caused by oxidative stress. This assay is known to give reliable information concerning the antioxidant ability of the tested compounds [12,13]. DPPH radical involves a hydrogen atom transfer process [14]. In this assay, the moderate antioxidant activity on DPPH radicals of lantadene A may be attributed to a direct role in trapping free radicals by donating hydrogen atom. Other anti-oxidant assays need to be undertaken to verify this point and to understand the full potential of lantadene A as an antioxidant agent.

### 2.2. Hydroxyl Radical Scavenging Activity

**Figure 3:**
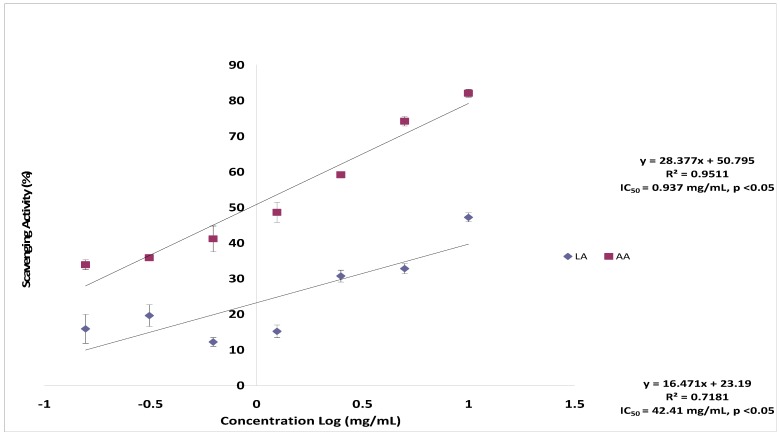
Scavenging effect of lantadene A on hydroxyl radicals compared to ascorbic acid.

The scavenging abilities of lantadene A on hydroxyl radical inhibition are shown in Figure 3. As we can see from Figure 3, lantadene A showed lower hydroxyl radical scavenging activity on hydroxyl radical. The maximum scavenging percentage of hydroxyl radical was 47.19 ± 1.26% at the concentration of 10.00 mg/mL. Furthermore, the IC_50_ value of lantadene A was 42.410 mg/mL (Figure 3), which was higher than the reference ascorbic acid (0.937 mg/mL), indicating impotent activity. Hydroxyl radicals are extremely strong reactive oxygen species, and there is no specific enzyme to defend against them in the human body [15]. For this reason, the discovery of some compounds with excellent hydroxyl radical scavenging ability would be significant for some illnesses induced by oxidative stress. In this study, lantadene A showed important hydroxyl radical inhibition activity which can be attributed to the combined effects of reducing power, donation of hydrogen atoms and scavenging of active oxygen. 

### 2.3. Superoxide Anion Radical Scavenging Activity

**Figure 4:**
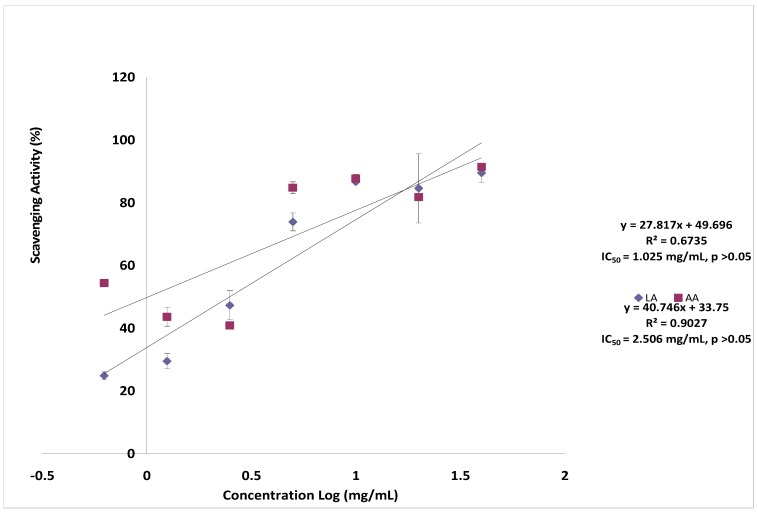
Scavenging effect of lantadene A on superoxide anion radical compared to ascorbic acid.

The effects of lantadene A on superoxide anion radical scavenging activity was estimated by the nitro blue tetrazolium (NBT) assay and the result is shown in Figure 4. Lantadene A showed a scavenging activity on the superoxide radicals in a dose dependent manner. The maximum scavenging percentage of superoxide radical was 89.38 ± 3.07% at the concentration of 40.0 mg/mL. Furthermore, the IC_50_ value of lantadene A was 2.506 mg/mL. Nonetheless, when compared to ascorbic acid (1.025 mg/mL), the superoxide radical-scavenging activity of lantadene A was found to be low, but comparable. Superoxide anion is a relatively weak oxidant, but it can generate more dangerous species, including singlet oxygen and hydroxyl radicals, which could cause tissue damage [16]. Hydrogen peroxide itself is not very reactive, but it may induce hydroxyl radicals, which would result in great damage to cells [17]. In this study, lantadene A showed potent scavenging activity on the superoxide anion radical. Hagerman *et al.* [18] have explained that high molecular weight and the proximity of many aromatic rings and hydroxyl groups are more important for the free radical-scavenging activity of bioactive compound. This indicates that presents of aromatic rings in lantadene A could be responsible for the good superoxide anion radical scavenging activity observed in this study. 

### 2.4. Nitric Oxide Scavenging Assay

**Figure 5:**
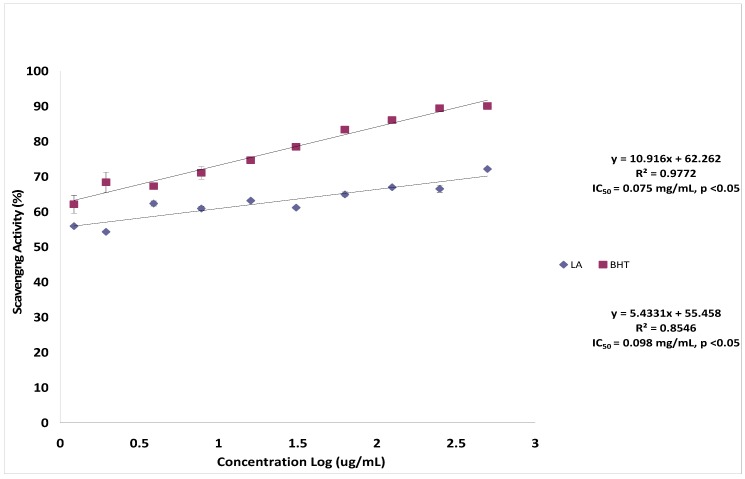
Scavenging effect of lantadene A on nitric oxide radicals compared with butylated hydroxytoluene (BHT).

The nitric oxide scavenging potential of lantadene A as compared with standard BHT is presented in Figure 5. In this assay, lantadene A showed potent nitric oxide scavenging activity in a dose dependent manner. The maximum scavenging percentage of superoxide radical was 72.12 ± 0.38% at the concentration of 500.00 µg/mL. Furthermore, the IC_50_ value of lantadene A was 98.00 µg/mL. Compared to BHT (75.00 µg/mL), the nitric oxide scavenging activity of lantadene A was found to be low but comparable. The role of nitric oxide in various disease states has attracted the attention of scientists worldwide. The nitric oxide does not interact with bioorganic macromolecules such as DNA or proteins directly. However, under aerobic conditions, the nitric oxide molecule is very unstable and reacts with the oxygen to produce intermediates such as NO_2_**·**, N_2_O_4_**·** and N_3_O_4_**·** and stable products like nitrate and nitrite [19,20] and peroxynitrite when reacted with superoxide [21,22] which is highly toxic to humans. The current study showed that the lantadene A may have the property to counteract the effect of nitric oxide formation and in turn may be of considerable interest in preventing the ill effects of excessive nitric oxide generation *in vivo* which deserves further study. The implications of this finding may be very significant for human health, since these drugs have been marketed as therapeutic agents and a couple of synthetic pentacyclic triterpenes derivatives also now undergoing clinical trials. Detailed *in vivo* studies are planned to explore the efficacy of lantadene A in ameliorating the NO induced damage.

### 2.5. Ferrous Ion Chelating Assay

**Figure 6:**
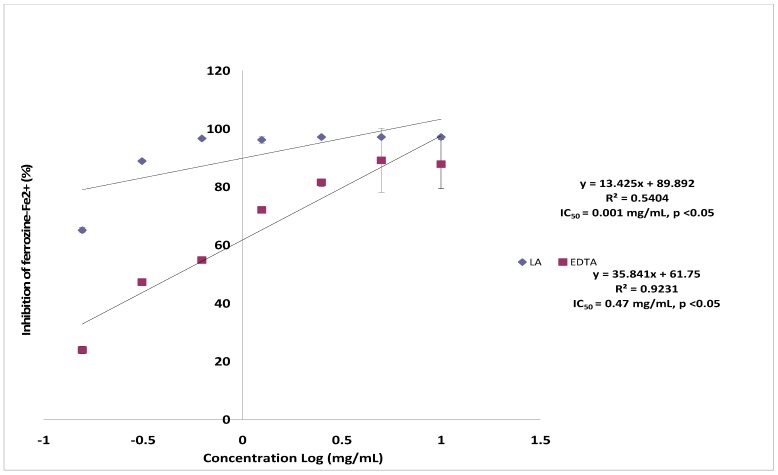
Ferrous ion chelating effect of lantadene A on ferrous ions compared to EDTA.

The chelating of Fe^2+^ by lantadene A was estimated by the method of Dinis *et al.* [23]. Ferrozine can quantitatively form complexes with Fe^2+^, however, in the presence of chelating agents, the complex formation is disrupted with the result that the red colour of the complex is decreased. Measurement of colour decrease, therefore, allows the assessment of the chelating activity of the coexisting chelator [24]. The result of ferrous ion chelating assay is presented in Figure 6. Lantadene A showed ferrous ion chelating activity in a dose dependent manner. The maximum ferrous ion chelating activity was 97.17 ± 0.18% at the concentration of 1.0 mg/mL. Furthermore, the IC_50_ value of lantadene A was 0.470 mg/mL. Nevertheless, when compared to EDTA (0.001 mg/mL), the ferrous ion chelating activity of lantadene A was found to be low. Iron chelators mobilize tissue iron by forming soluble, stable complexes that are then excreted in the feces and/or urine. Chelation therapy reduces iron-related complications and thereby improves quality of life and overall survival [25,26]. The accumulation of toxic quantities of iron causes tissue damage and leads to various complications in human. Therefore, lantadene A can be observed as a potent ferrous-chelating source worthy of further investigation.

### 2.6. Reducing Power

**Figure 7:**
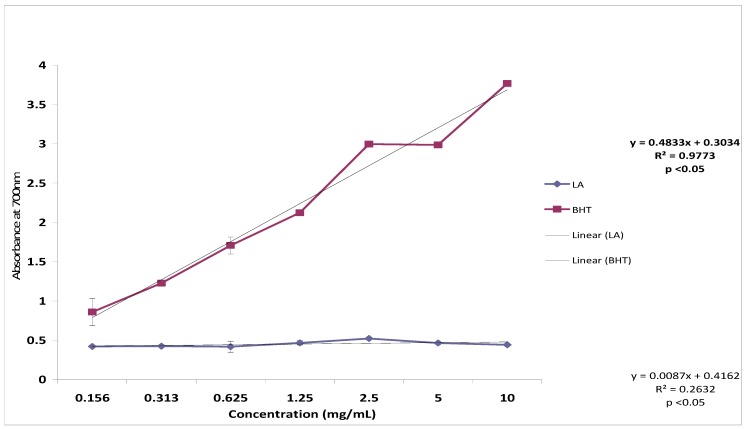
Reducing power of lantadene A compared to butylated hydroxytoluene (BHT).

Figure 7 shows the reductive capabilities of lantadene A in comparison to the standard, BHT. It was found that the reducing power was not increased with increasing concentration of lantadene A. In the present study, lantadene A showed no reducing ability and was less than the standard, BHT. The presence of reductants (antioxidant compounds) would result in reduction of the Fe^3+^/ferric cyanide complex to the ferrous form. The Fe^2+^ could therefore, be monitored by measuring the formation of Perl’s Prussian blue with absorbance at 700 nm. The compounds with strong reducing power had a stronger peroxide reducing ability. Hence, the current study proved that lantadene A had a weaker peroxide reducing ability.

### 2.7. Toxicity and Possible Molecular Mechanisms of Lantadene A Action

**Figure 8:**
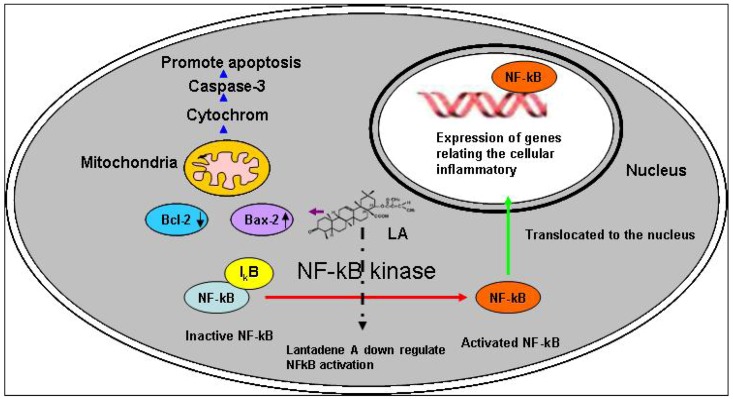
Proposed model of molecular mechanism of lantadene A (LA)-induced apoptosis and down-regulation of NFkB activation in cells.

In order to understand the molecular mechanisms of lantadene A’s action, we recommend future studies should focus on whether lantadene A suppressed pro-inflammatory transcription factors such as transcription factor nuclear factor-*k*B (NF-*k*B) and NF-IL-6 or activating protein-1 (AP-1). The transcription factor NFkB regulates the expression of a wide range of genes involved in immune response, inflammation, and acute phase response, as well as several viral genes. Recent evidence also suggests that NFkB is involved in carcinogenesis and apoptosis [27]. Moreover, various kinds of antioxidants have been reported to down-regulate NFkB activation in a wide range of cell types [28] (Figure 8). Furthermore, lantadene A treatment may up-regulates Bax, down-regulates Bcl-2 expression, promotes Bax translocation to mitochondria, activates mitochondria-mediated apoptotic pathway, which in turn causes the release of caspase-3, and promote cell apoptosis which deserved further study (Figure 8). Our previous results on *L. camara*’s leaf extracts which consist of lantadene A, did not showed any apparent toxicity towards Vero cells with an IC_50_ value of 319.37 ± 99.80 µg/mL at 72 h. However, the results of oral acute toxicity study on *L. camara* leaf showed a pro-toxic effect. Hence, further toxicity studies are needed to determine the acute and chronic oral toxicity effects of lantadene A on animal fetus, pregnant animals, and their reproductive capacity, to complete the safety profile of this compound which should be very useful for any future *in vivo* or clinical study of this compound [29].

## 3. Experimental

### 3.1. Preparation of the Lantadene A

Lantadene A was purchased from local chemicals agents.

### 3.2. DPPH Radical-Scavenging Assay

The stable 1,1-diphenyl-2-picrylhydrazyl radical (DPPH) was used for determination of free radical scavenging activity of the extract [30]. The reaction mixture contained 50 μL of different concentrations of lantadene A and 5 mL of 0.04% (w/v) solution of DPPH in 80% methanol. After 30 min at room temperature, the absorbance was recorded at 517 nm using spectrophotometer (HITACHI U-1900 spectrophotometer 200V). The commercial known antioxidant, butylated hydroxytoluene (BHT) was used as a positive control. The experiment was performed in triplicate. The percentage of the DPPH free radical was calculated using the following equation:



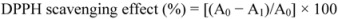



where A_0_ was the absorbance of the control and A_1_ was the absorbance in the presence of the lantadene A or positive control). The IC_50_ (concentration providing 50% inhibition) values were calculated use the dose inhibition curve in linear range by plotting the lantadene A concentration versus the corresponding scavenging effect.

### 3.3. Reducing power Assay

The reducing power of lantadene A was determined according to the method of Yen and Chen [31] with a slight modification. Different concentrations of lantadene A (0.5 mL) were mixed with phosphate buffer (2.5 mL, 0.2 M, pH 6.6) and potassium ferricyanide [K_3_Fe(CN)_6_] (2.5 mL, 1%). The mixture was incubated at 50 °C for 20 min. A portion (2.5 mL) of trichloroacetic acid (10%) was added to the mixture to terminate the reaction, which was then centrifuged at 3000 rpm for 10 min. The upper layer of solution (2.5 mL) was mixed with distilled water (2.5 mL) and FeCl_3_ (0.5 mL, 0.1%), the reaction mixture was left for 10 min at room temperature and the absorbance was measured at 700 nm. All tests were performed six times. A higher absorbance of the reaction mixture indicated greater reducing power. BHT was used as a positive control.

### 3.4. Hydroxyl Radical Scavenging Assay

The capacity to scavenge hydroxyl radicals was measured according to a modification of the method of Halliwell *et al.* [32]. The hydroxyl radicals generates by iron- ascorbate-EDTA-H_2_O_2_, which then attack deoxyribose to form thiobarbituric acid reactive substances (TBARS) which yield pink chromogen at low pH while heating with trichloroacetic acid (TBA). The hydroxyl scavengers, from leaves lantadene A compete with deoxyribose for hydroxyl radicals and decreases TBARS lead to reduction in formation of pink chromogen [33]. The reaction mixture contained 4 mM deoxyribose, 0.3 mM ferric chloride, 0.2 mM EDTA, 0.2 mM ascorbic acid, 2 mM H_2_O_2_ and various concentration of test compounds was added. The tubes were capped tightly and incubated for 30 min at 37 °C. Then the reaction mixture was added with 0.4 mL of 5% TBA and 0.4 mL of 1% TBA. The reaction mixture was kept in boiling water bath for 20 min. The intensity of pink chromogen was measured spectrophotometrically at 532 nm against blank sample. Ascorbic acid was used as a positive control. All tests were performed in triplicate. The hydroxyl radical scavenging activity of lantadene A reported as % inhibition of deoxyribose degradation and was calculated by following equation: 



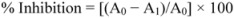



where A_0_ was the absorbance of the control and A_1_ was the absorbance in the presence of the lantadene A or positive control.

### 3.5. Nitric Oxide Scavenging Assay

At physiological pH, nitric oxide generated from aqueous sodium nitroprusside (SNP) solution interacts with oxygen to produce nitrite ions which were measured by Griess Illosvoy reaction [34]. The reaction mixture contained SNP (10 mM), phosphate buffer saline (pH 7.4) and different concentrations of lantadene A and was incubated for 180 min at 25 °C. Then samples from the above were reacted with Griess reagent (1% sulphanilamide, 0.1% naphthylethylenediamine dichloride (NED) and 3% phosphoric acid) and mixture was incubated for 30 min at 25 °C. The pink chromophore formed during diazotization of nitrite ions with sulphanilamide and subsequent coupling with NED was measured spectrophotometrically at 540 nm against blank sample. BHT was used as a positive control [35]. All tests were performed in triplicate. The nitric oxide radicals scavenging activity was calculated according to the equation:



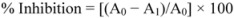



where A_0_ was the absorbance of the control and A_1_ was the absorbance in the presence of the lantadene A or positive control. 

### 3.6. Ferrous Ion Chelating Assay

The chelating activity of the lantadene A for ferrous ions (Fe^2+^) was measured according to the method of Dinis *et al* [23]. Briefly, 0.5 mL different concentration of lantadene A was added to a solution of 2 mM FeCl_2_ (0.05 mL). The reaction was initiated by the addition of 5 mM ferrozine (0.2 mL). The mixture was shaken vigorously and left at room temperature for 10 min. Ferrozine reacted with the divalent iron to form stable magenta complex species that were very soluble in water. Absorbance of the solution was then measured spectrophotometrically at 562 nm. The percentage inhibition of ferrozine-Fe^2+^ complex formation by lantadene A extract was calculated as:



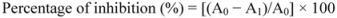



where A_0_ was the absorbance of the control, and A_1_ was the absorbance of the lantadene A or EDTA (positive control).

### 3.7. Determination of Superoxide Anions Scavenging Activity

The superoxide anions generated by phenazine methosulphate (PMS) and nicotinamideadanine dinucleotidphosphate, reduced form (NADPH), was detected by the reaction with 2,2'-di-*p*-nitrophenyl-5,5'-diphenyl-(3,3'-dimethoxy-4,4'-diphenylene) di-tetrazolium chloride (nitro blue tetrazolium-NBT) [36]. Reaction mixture contained 1 mL samples (different concentration), 1 mL of NBT solution (312 μM prepared in phosphate buffer, pH −7.4) and 1 mL of NADH solution (936 μM prepared in phosphate buffer, pH −7.4). Finally, the reaction was accelerated by adding 100 μL PMS solution (120 μM prepared in phosphate buffer, pH −7.4) to the mixture. The reaction mixture was incubated at 25 °C for 5 min and absorbance at 560 nm was measured against methanol as control. Percentage inhibition and IC_50_ value was calculated using the formula: 



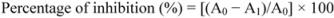



where A_0_ was the absorbance of the control, and A_1_ was the absorbance of the lantadene A or ascorbic acid (positive control).

## 4. Conclusions

Modern medicine has extensively used plant-derived compounds such as lantadene A for the treatment of various ailments affecting human health. Although many *in vitro* assays were used in this study to evaluate the antioxidant activity of lantadene A, the results obtained showed totally different results for the different assays. This finding proves that some compounds potentiate a desired antioxidant activity, while others do not reinforce the same activity. Therefore, compounds with the common desired activities and varied undesirable activities are selected so that the final formulation will have a concentrated desired activity and the undesired activities will be diluted or absent altogether. The mechanism of action of lantadene A may differ in many respects for the different free radicals. It can be characterized as a polyvalent action. Further, it has also been observed that in such formulations, certain other compounds may be of help in enhancing the potency of the active compounds resulting in an additive or synergistic positive effect, which in its final total provides immense benefits to the patient [37]. These findings show that the lantadene A possesses antioxidant activity with different mechanism of action towards different free radicals. Since the lantadene A is a very popular drug in modern medicine, it is a promising candidate for use as an antioxidant and hepatoprotective agent. Detailed *in vivo* studies are planned to explore the efficacy of lantadene A in ameliorating free radical induced damage.
